# Predictive analytics for cardio-thoracic surgery duration as a stepstone towards data-driven capacity management

**DOI:** 10.1038/s41746-023-00938-0

**Published:** 2023-11-07

**Authors:** Mariana Nikolova-Simons, Rikkert Keldermann, Yvon Peters, Wilma Compagner, Leon Montenij, Ymke de Jong, R. Arthur Bouwman

**Affiliations:** 1 Philips Research, Eindhoven, the Netherlands; 2 Catharina Hospital, Eindhoven, the Netherlands; 3 Eindhoven University of Technology, Department of Electrical Engineering, Eindhoven, the Netherlands

**Keywords:** Health services, Health care

## Abstract

Effective capacity management of operation rooms is key to avoid surgery cancellations and prevent long waiting lists that negatively affect clinical and financial outcomes as well as patient and staff satisfaction. This requires optimal surgery scheduling, leveraging essential parameters like surgery duration, post-operative bed type and hospital length-of-stay. Common clinical practice is to use the surgeon’s average procedure time of the last N patients as a planned surgery duration for the next patient. A discrepancy between the actual and planned surgery duration may lead to suboptimal surgery schedule. We used deidentified data from 2294 cardio-thoracic surgeries to first calculate the discrepancy of the current model and second to develop new predictive models based on linear regression, random forest, and extreme gradient boosting. The new ensamble models reduced the RMSE for elective and acute surgeries by 19% (0.99 vs 0.80, *p* = 0.002) and 52% (1.87 vs 0.89, *p* < 0.001), respectively. Also, the elective and acute surgeries “behind schedule” were reduced by 28% (60% vs. 32%, *p* < 0.001) and 9% (37% vs. 28%, *p* = 0.003), respectively. These improvements were fueled by the patient and surgery features added to the models. Surgery planners can benefit from these predictive models as a patient flow AI decision support tool to optimize OR utilization.

## Introduction

Worldwide, healthcare organizations (HCOs) are facing challenges due to an increased demand for care by an aging population and increased multi-morbidity^1–3^. This problem is exacerbated in the long term due to an increased shortage of healthcare professionals^4,5^ that cannot be solved by increasing healthcare budgets^6,7^. The necessity to improve healthcare systems using innovative strategies is high and health policymakers are proposing both—cost-containment and production improvement policies^8,9^. While cost-containment strategies ensure short-term savings they cannot guarantee long-term results^8^. Therefore, production improvement strategies utilizing capacity efficiently are key for long-term solutions^10–13^.

Effective capacity management strategies are based on understanding the barriers to streamlining hospital patient flow and associated root causes as described in several systematic reviews^14–17^. The most recent review by Ahlin et al.^18^ went a step further. First, it explored which factors are preventing swift patient throughput at hospitals and second, it synthesized these factors into main barriers and underlying root causes. The main barriers were long lead times and inefficient coordination during the patient transfer process, caused by inadequate staffing, lack of standards, insufficient operational planning, and a lack of IT support.

Capacity management strategies can benefit from digital innovations^19^. Artificial intelligence (AI) can power digital medicine clinically via better disease surveillance, improved diagnosis, and novel treatments^20^, as well as operationally via improved capacity utilization. The Catharina Hospital in Eindhoven, the Netherlands, is pioneering on efficient use of capacity resources and value-based healthcare^21–23^. The hospital provides outpatient and inpatient services for up to 415,000 patients annually with a workforce of ~400 physicians (attendings and residents) and 1250 nurses. It comprises 400 beds and 20 operation rooms with more than 16,000 surgeries performed annually. The Catharina Hospital specializes in cardio-vascular and oncology care^24^.

**Fig. 1:**
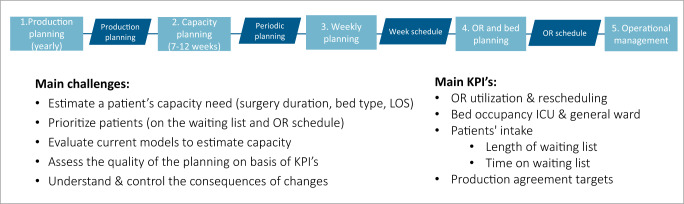
Catharina’s capacity management in surgery planning. Provides an overview of Catharina’s production planning, main capacity challenges, and key performance indicators (KPIs).

An overview of production planning, main capacity challenges, and key performance indicators (KPIs) is depicted in Fig. 1. Given the challenges the hospitals are facing, efficient planning and utilization of capacity resources such as operation rooms (OR), intensive care unit (ICU), post anesthesia care unit (PACU), and the general ward is imperative. To ensure their timely availability for patients and staff, surgery and bed planners are using predictions of surgery duration, post-OR bed type (ICU/PACU/general ward), and hospital length-of-stay.

This study focuses on predictive models for cardio-thoracic surgery duration as a stepstone towards data-driven capacity management. The current model to estimate the surgery duration of a patient with a surgical procedure X is based on 2 steps: (1) the surgeon’s average procedure X time of the last 10 patients and (2) a manual correction to account for patient’s specific characteristics if needed. While this model is simple to understand, the discrepancy (delta) between the actual and planned surgery duration can be substantial and cause suboptimal surgery scheduling. This leads to inefficient OR utilization, surgery rescheduling, long waiting lists, staff overtime, and high workload.

The study objective was (i) to evaluate the performance of the current surgery duration model used in clinical practices, (ii) to develop and validate an enhanced predictive model and (iii) to get insight into which patient and surgery characteristics are key features in the model development.

## Results

### Surgery inclusion criteria

**Fig. 2:**
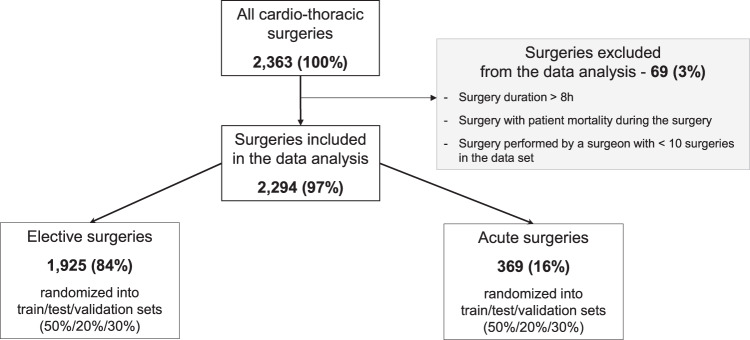
Surgery flowchart. Summarizes the included and excluded elective and acute surgeries used to develop and evaluate the predictive models of surgery duration—training, test, and validation sets.

The surgeries included and excluded in the analysis are illustrated in Fig. 2. In summary, 2363 cardio-thoracic surgeries were performed in the Catharina hospital on 2144 patients older than 18 years in the period Dec 2018—Feb 2020, prior to the COVID-19 pandemic. We excluded 69 (out of 2363; 3%) surgeries according to the exclusion criteria mentioned in the Methods section and Fig. 2. Hence, 2294 (97%) surgeries were included in the analyses, performed on 2098 patients. Thus, 165 patients (out of 2098; 8%) had multiple surgeries, e.g., CABG followed by a resternotomy, both included in the analysis. These 165 patients included 44 patients with at least two elective surgeries, 38 patients with at least two acute surgeries, and 83 patients with one elective and one acute surgery. We refer to the total set as the overall cohort. Most of the surgeries were elective—1925 (out of 2294; 84%) vs. 369 (out of 2294; 16%) acute.

### Surgery and patient characteristics

The characteristics of the three surgery cohorts—elective, acute, overall—are summarized in Tables 1 and 2. There were statistically significant differences between all characteristics of the elective and acute cohorts, except gender and type of surgeon. The patients characteristics with the highest prevalence in the overall surgery cohort were the age category 60–74 (54%), male (75%), overweight (39%), ASA (American Society of Anesthesiologists) score = 3 (44%), medications category either 1–5 (27%) or 6–10 (26%) and normal creatinine level (44%). The most common surgery procedure in the overall cohort was CABG (49%, Coronary Artery Bypass Graft) followed by AVR (14%, Aortic Valve Replacement). Only 17% of overall surgeries had at least two procedures performed during the same surgery. Nearly two-third of overall surgeries were performed by attending physicians. The three post-OR bed types (ICU, PACU, general ward) were with similar utilization. The target variable—surgery duration—had an average value of 3.5 h in the overall cohort.

**Table 1:** Patient characteristics by surgery cohort.

Cardio-thoracic surgeries				
Characteristics	Elective *n* = 1925	Acute *n* = 369	Overall *n* = 2294	*p* value
Patients, *n* (%)	1878	323	2098	
Patients with multiple surgeries, *n* (%)	44 (2)	38 (12)	165 (8)	<0.001
Age category, *n* (%)				<0.001
18–29	50 (2.6)	17 (4.6)	67 (2.9)	
30–44	37 (1.9)	20 (5.4)	57 (2.5)	
45–59	374 (19.4)	86 (23.3)	460 (20.1)	
60–74	1061 (55.1)	175 (47.4)	1236 (53.9)	
75+	403 (20.9)	93 (19.2)	474 (20.7)	
Gender, male, *n* (%)	1449 (75.3)	276 (74.8)	1725 (75.2)	0.898
BMI category, *n* (%)				<0.001
Normal	483 (25.1)	65 (17.6)	548 (23.9)	
Overweight	816 (42.4)	83 (22.5)	899 (39.2)	
Obese	462 (24.0)	42 (22.5)	504 (22.0)	
Unknown	164 (8.5)	179 (48.5)	343 (15.0)	
ASA category, *n* (%)				<0.001
1	30 (1.6)	3 (0.8)	33 (1.4)	
2	114 (5.9)	12 (3.3)	126 (5.5)	
3	977 (50.8)	34 (9.2)	1011 (44.1)	
4+	565 (29.4)	86 (23.3)	651 (28.4)	
Unknown	239 (12.4)	234 (63.4)	473 (20.6)	
Medications category, *n* (%)				<0.001
1–5	548 (28.5)	79 (21.4)	627 (27.3)	
6–11	527 (27.4)	77 (20.9)	604 (26.3)	
11+	151 (7.8)	21 (5.7)	172 (7.5)	
Unknown	699 (36.3)	192 (52.0)	891 (38.8)	
Creatinine category, *n* (%)				<0.001
Normal	899 (46.7)	113 (30.6)	1012 (44.1)	
Moderate decreased	738 (38.3)	91 (24.7)	829 (36.1)	
Severe decreased	138 (7.2)	35 (9.5)	173 (7.5)	
Unknown	150 (7.8)	130 (35.2)	280 (12.2)	

*p* value of Student *t* tests/Mann–Whitney *U* tests for normally/non-normally distributed continuous variables and Pearson Chi-square tests for categorical variables.

**Table 2:** Surgery characteristics by surgery cohort.

Cardio-thoracic surgeries				
Characteristics	Elective *n* = 1925	Acute *n* = 369	Overall *n* = 2294	*p* value
Surgery urgency, acute, *n* (%)	0 (0.0)	369 (100.0)	369 (16.1)	<0.001
Procedure cluster, *n* (%)				<0.001
Lungs	173 (9.0)	62 (16.8)	235 (10.2)	
Pectus	67 (3.5)	2 (0.5)	69 (3.0)	
Vascular	14 (0.7)	29 (7.9)	43 (1.9)	
Heart				
CABG*	1023 (53.1)	105 (28.5)	1128 (49.2)	
CABGtotal	60 (3.1)	7 (1.9)	67 (2.9)	
AVR**	313 (16.3)	15 (4.1)	328 (14.3)	
AVRaorta	36 (1.9)	3 (0.8)	39 (1.7)	
MVP***	79 (4.1)	4 (1.1)	83 (3.6)	
MVR****	33 (1.7)	6 (1.6)	39 (1.7)	
Resternotomy	3 (0.2)	84 (22.8)	87 (3.8)	
Heart others	76 (3.9)	26 (7.0)	102 (4.5)	
Sternum refixation	39 (2.0)	10 (2.7)	49 (2.1)	
Others	9 (0.5)	16 (4.3)	25 (1.1)	
Procedures during surgery, *n* (%)				0.002
Single	1568 (81.5)	328 (88.9)	1896 (82.7)	
Double	298 (15.5)	33 (8.9)	331 (14.4)	
Multiple (3+)	59 (3.1)	8 (2.2)	67 (2.9)	
Surgeon type, attending, *n* (%)	1234 (64.1)	234 (63.4)	1468 (64.0)	0.847
Post-OR bed type, *n* (%)				<0.001
General ward	459 (23.8)	284 (77.0)	743 (32.4)	
ICU	647 (33.6)	71 (19.2)	718 (31.3)	
PACU	819 (42.5)	14 (3.8)	833 (36.3)	
Surgery duration, mean (SD) hours	3.63 (1.14)	2.81 (1.66)	3.50 (1.28)	<0.001

*CABG coronary artery bypass graft, **AVR aortic valve replacement, ***MVP mitral valve plastic, ****MVR mitral valve replacement, *p* value of Student *t* tests/Mann–Whitney *U* tests for normally/non-normally distributed continuous variables and Pearson Chi-square tests for categorical variables.

Further, similar analysis was performed on the development, test, and validation sets. No statistically significant differences were found between the characteristics, confirming that the randomization ensured set similarity.

### Evaluation of the current model of surgery duration

**Fig. 3:**
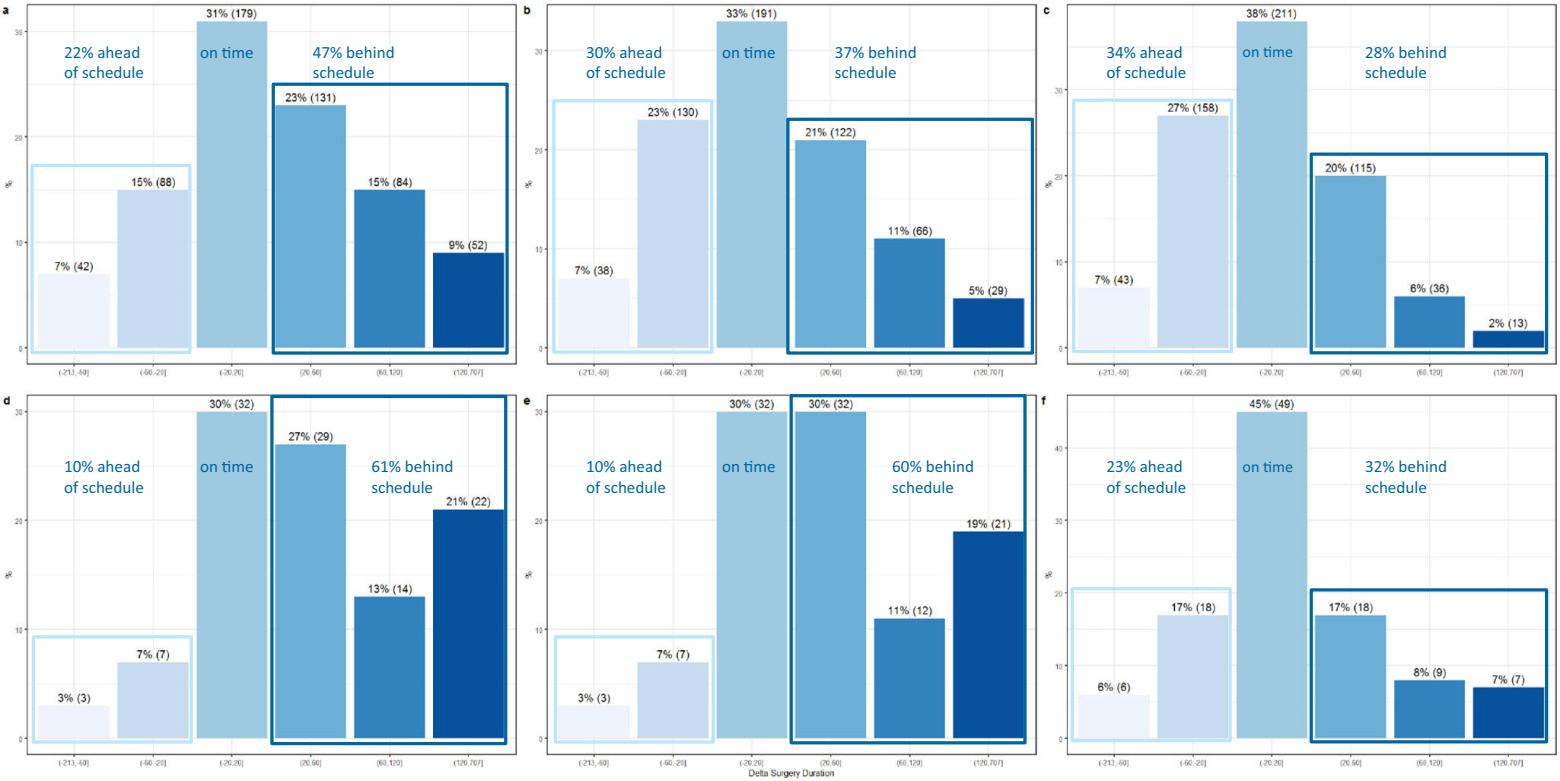
Performance of the current and the ensemble models of surgery duration—customized errors on the validation set. Visualizes the delta between real and planned elective **a**–**c** and acute **d**–**f** surgery duration: **a**, **d** current model without surgeon correction; **b**, **e** current model with surgeon correction; **c**, **f** new ensemble models.

We evaluated the performance of the current model of surgery duration using the root mean square error (RMSE) and the mean absolute error (MAE) for both elective (RMSE_elective_ = 0.99 and MAE_elective_ = 0.71), and acute surgeries (RMSE_acute_ = 1.87 and MAE_acute_ = 1.22), respectively, see Table 4. While RMSE and MAE are common errors for regression models in data analysis, they do not provide any scheduling insights into OR utilization. Therefore, we clustered the surgeries with respect to their differences between real and planned surgery duration into meaningful categories for OR utilization, namely surgeries “on time”, “behind schedule” and “ahead of schedule”, and named them customized errors, see Table 5 and Fig. 3. The analyses showed that in 43% and 19% of all elective and acute surgeries, respectively, the average surgical procedure duration (step 1 of the current model) was manually corrected by the surgeons (step 2 of the current model). After the correction, see Fig 3b and e, (1) 37% of all elective surgeries and 60% of all acute surgeries were “behind schedule”, (2) 33% of all elective surgeries and 30% of all acute surgeries were “on time”, and (3) 30% of all elective surgeries and 10% of all acute surgeries were “ahead of schedule”. These discrepancies were the rationale for developing improved ML predictive models that leverage additional patient and surgery characteristics.

### ML predictive models of surgery duration development

Table 3 provides an overview of the patient and surgery characteristics used to develop the ML predictive models for surgery duration. The features were extracted from the surgery request form and the pre-operative screening recorded in the EHR prior to the patients’ surgery. The feature selection had two steps based on univariate and multivariate analyses, respectively, as indicated in Table 3.

**Table 3:** Features based on patient and surgery characteristics.

Features		Examples	Univariate analysis	Multivariate analysis
Age	Categorical	[18–29], [30–44], [45–69], …	√	×
Gender	Categorical	Male vs. female	×	×
BMI (body mass index)	Categorical	Underweight, normal, overweight, obese	×	×
ASA score	Categorical	1, 2, 3, 4, 5	√	√
Medications	Categorical	[1–5], [6–10], [11–15], 16++	√	√
Creatinine levels	Categorical	Renal failure, severe decrease, moderate decrease, normal	√	√
Surgeon ID	Categorical, unclustered	Surgeon_6791, Surgeon_86	√	√
	Categorical, clustered	Hierarchical clustering - Surgeon_C1, Surgeon_C2		
Surgeon type	Categorical	Attending vs. resident	√	×
Surgery procedures (Px)	Categorical, unclustered	CABG*, AVR**	√	√
	Categorical, clustered	Medical—CABG, AVR, Lungs, etc.; Hierarchical—Px_C1, Px_C2		
Number of procedures	Categorical	Single, double, multiple (≥3)	√	√
Surgery urgency	Categorical	Acute vs. elective	√	√
Post-OR bed type estimate	Categorical	ICU, PACU, general ward	√	√

*CABG coronary artery bypass graft, **AVR aortic valve replacement, *V* selected, *x* excluded.

**Fig. 4:**
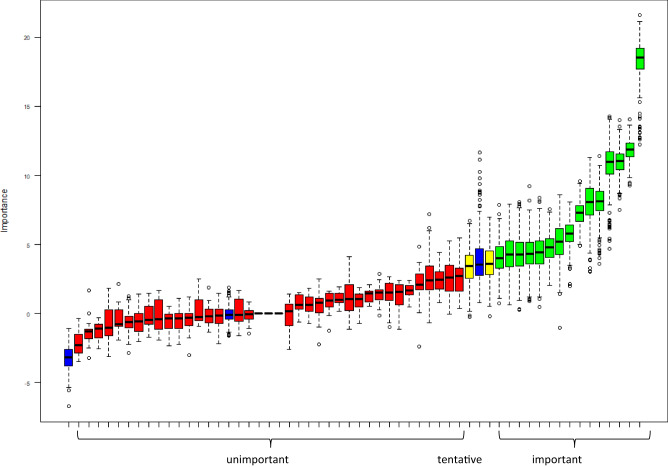
Feature selection. Depicts the output of Boruta algorithm—features on the *x* axis clustered into important (green), tentative (yellow), and unimportant (red) according to their importance on the *y* axis.

The univariate analysis revealed that gender and BMI are not statistically significant predictors (*p* > 0.05). However, BMI is a significant factor in the anesthesiologist/surgeon clinical evaluation before and during the pre-operative screening and has another weight for elective than acute surgeries. The remaining features went through the multivariate analysis using Boruta algorithm^25^. It clustered the features into important, tentative, and unimportant, see Fig. 4, where each boxplot corresponds to a category of a feature from Table 3. For example, five of the box plots in Fig. 4 represent the importance of ASA score categories 1, 2, 3, 4, and 5 as features. Examples of unimportant features were the surgeon type (attending vs. resident) and age. The important and tentative features were selected to develop the predictive models using three ML techniques—linear regression (LM), random forest (RF), and extreme gradient boosting (XGBoost; abbreviated as GB). We trained the models on the three surgery cohorts—elective, acute, and overall surgeries. In the remainder of the paper, we dropped the models trained on the overall surgeries since they were outperformed by the dedicated models trained on elective-only and acute-only surgeries. The outperformance is explained by the statistically significant differences between the elective and acute cohorts shown in Tables 1 and 2.

**Fig. 5:**
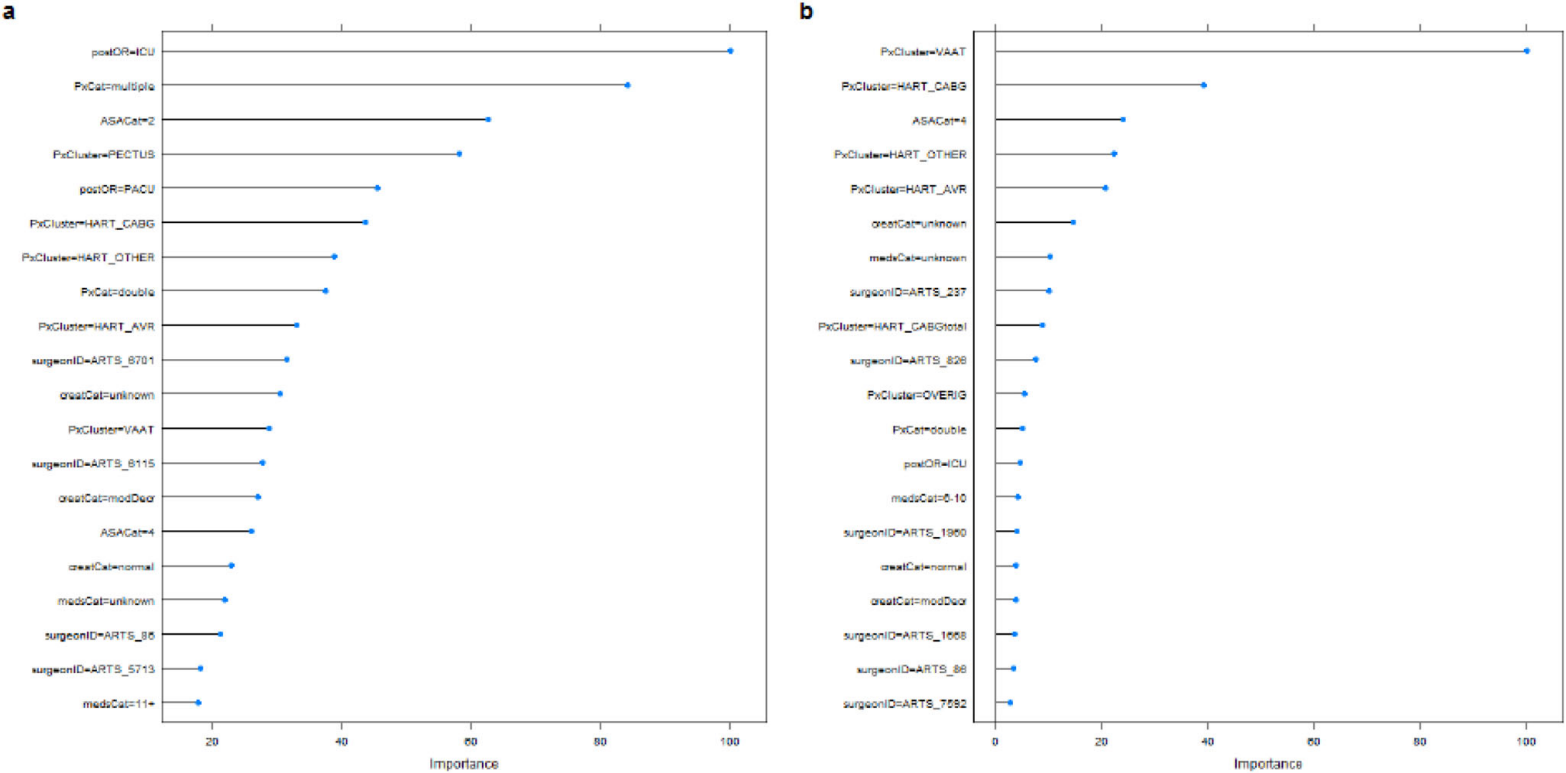
Feature importance. Visualizes the feature importance of the RF models for **a** elective and **b** acute cardio-thoracic surgery.

Figure 5a, b illustrates the importance of the top 20 features of the RF models. Note that all features are defined during pre-operative screening. The 3 most important predictors for the duration of elective surgery were (1) the anesthesiologist estimate of post-OR bed type being ICU rather than PACU or general ward, (2) the multiple number of procedures during the surgery, e.g., CABG and AVR, and (3) the ASA score being at least 2^26^. The three most important predictors for the duration of acute surgery were (1) the surgery procedure being in the vascular cluster compared to other procedure clusters, (2) the surgery procedure being in the CABG cluster, and (3) the ASA score being at least 4. The top 20 predictors of the LM and GB models were similar although their ranking was different. More insights into the feature importance are provided in the Discussion section.

### Evaluation of the ML models of surgery duration

Table 4 shows the predictive models performance in terms of RMSE and MAE on the validation data sets and the % error reduction compared to the current model. The latter includes both the correction by a surgeon on top of the average procedure time estimate. The GB model for elective cardio-thoracic surgery duration showed the best RMSE and MAE error reduction compared to the current model: −19% for RMSE (from 0.99 to 0.80, *p*=0.002) and −14% for MAE (from 0.71 to 0.61, *p*=0.005). The GB model for acute cardio-thoracic surgery duration also showed the best RMSE error reduction compared to the current model: −52% (from 1.87 to 0.89, *p*<0.001). However, the RF model for acute cardio-thoracic surgery duration was slightly better than the GB model in reducing the MAE error: −50% (from 1.22 to 0.61, *p*<0.001).

**Table 4:** Model performances—RMSE, MAE errors on the validation set.

Models for Cardio-Thoracic surgery		Elective*		Acute**	
		RMSE [95% CI] % reduction, p***	MAE [95% CI] % reduction, *p*	RMSE [95% CI] % reduction, *p*	MAE [95% CI] % reduction, *p*
Current****		0.99 [0.94, 1.05]	0.71 [0.67, 0.75]	1.87 [1.72, 2.25]	1.22 [1.11, 1.46]
New ML models	LM	0.84 [0.79, 0.89] −15%, *p* = 0.01	0.65 [0.61, 0.69] −9%, *p* = 0.09	0.92 [0.82, 1.07] −50%, *p* < 0.001	0.66 [0.58, 0.76] −46%, *p* < 0.001
	RF	0.83 [0.78, 0.88] −16%, *p* = 0.007	0.62 [0.59, 0.66] −12%, *p* = 0.02	0.92 [0.81, 1.06] −50%, *p* < 0.001	0.61 [0.53, 0.70] −50%, *p* < 0.001
	GB	0.80 [0.76, 0.85] −19%, *p* = 0.002	0.61 [0.57, 0.65] −14%, *p* = 0.005	0.89 [0.79, 1.03] −52%, *p* < 0.001	0.62 [0.54, 0.71] −49%, *p* < 0.001
	Ensemble	0.79 [0.75, 0.84] −20%, *p* = 0.001	0.60 [0.57, 0.64] −15%, *p* = 0.004	0.91 [0.86, 1.13] −51%, *p* < 0.001	0.60 [0.56, 0.73] −50%, *p* < 0.001

*RMSE* root mean square error, *MAE* mean absolute error, *%reduction* % error reduction compared to the current model, *ML* machine learning, *LM* linear regression model, *RF* random forest, *GB* extreme gradient boosting.

*ML predictive models trained on elective-only surgeries; **ML predictive models trained on acute-only surgeries; ***p value of Student *t* tests comparing to the current model; ****current model incl. both steps of average procedure time and a correction by a surgeon.

Table 5 shows the predictive models’ performance in terms of customized errors on the validation data sets.

**Table 5:** Model performances—customized errors on the validation set.

Models for Cardio-Thoracic surgery			Ahead of schedule		On time	Behind schedule;			P***
			(--, –60] min	(−60, −20] min	(−20, 20] min	(20, 60] min	(60, 120] min	(120, ++) min	
Elective*	Current****		7%	23%	33%	21%	11%	5%	
	New ML models	LM	9%	27%	33%	20%	9%	2%	0.019
		RF	7%	25%	37%	22%	7%	2%	0.017
		GB	7%	26%	35%	23%	7%	2%	0.016
		Ensemble	7%	27%	38%	20%	6%	2%	0.002
Acute**	Current****		3%	7%	30%	30%	11%	19%	
	New ML models	LM	5%	29%	32%	20%	12%	2%	<0.001
		RF	3%	24%	39%	22%	8%	4%	<0.001
		GB	4%	31%	37%	16%	9%	3%	<0.001
		Ensemble	6%	17%	45%	17%	8%	7%	<0.001

*ML* machine learning; *LM* linear regression model; *RF* random forest; *GB* extreme gradient boosting; ensemble models - LM + RF + GB stacked by LM for elective and LM + GB stacked by RF for acute

*ML models trained on elective-only surgeries; **ML models trained on acute-only surgeries; ***p value of Pearson Chi-square comparing to the current model; ****current model includes both steps of average procedure time and correction by the surgeon.

Summing up, all three models LM, RF, and GB had a statistically significant error reduction compared to the current model (see Tables 4 and 5) with a slight outperformance of the GB/RF models compared to the LM model. To further improve the model performance, we created multiple ensemble models using the LM, RF, and GB predictions on the test data sets.

The best predictive model of elective cardio-thoracic surgery duration, according to the customized errors, was an ensemble model—LM + RF + GB predictions stacked by LM (see Table 5). It reduced the number of surgeries “behind schedule” by −9% (from 37 to 28%) and boosted the surgery “on time” by +5% (from 33 to 38%), see also Fig. 3b, c. The number of surgeries “ahead of schedule” was increased by +4% (from 30 to 34%).

The best predictive model of acute cardio-thoracic surgery duration, according to the customized errors, was an ensemble model, namely LM + GB predictions stacked by RF (see Table 5). It reduced the number of surgeries “behind schedule” by −28% (from 60 to 32%) and boosted the surgery “on time” by +15% (from 30 to 45%), see also Fig. 3e, f. The number of surgeries “ahead of schedule” was increased by +13% (from 10 to 23%) and this is the price we paid.

## Discussion

The analyses revealed a couple of key findings. First, the discrepancy between the real and planned surgery durations in the current clinical practice is substantial. In cardio-thoracic surgery 37% of all elective surgeries and 60% of all acute surgeries were “behind schedule”, see Fig. 3b, e. Similar percentages were reported by Rozario^27^ on aggregated OR level—ORs were overtime 48% of the time. In both studies, as well as in^27^, the current planned surgery duration is the surgeon’s average procedure time of the last 10 patients. In our study, 43% and 19% of the elective and acute average procedure times, respectively, were corrected manually by surgeon. Whilst the corrections for elective surgeries reduced “behind schedule” surgeries from 47 to 37%, see Fig. 3a, b, this reduction for acute surgeries was minimal - from 61 to 60%, see Fig. 3d, e. The current model is surgeon- and procedure-specific with an optional manual patient-specific correction that cannot completely resolve the discrepancy. Furthermore, the last 10 patients are not representative of the next individual patient to be planned. Taking an average of only 10 patients can be inaccurate due to deviation induced by the small sample size and outlier skewness. In contrast, the ML models we developed included (1) 15 months of data cleaned from extreme outliers, so that the outliers’ effect is minimal and (2) more patient and surgery characteristics than the surgeon and the procedure only. Both powered the reduction of discrepancy between the real and planned surgery durations.

The second key finding is that the features quantifying and qualifying the surgery and patient complexity are the most important features of our predictive models (see Fig. 5). For example, type and number of procedures during the surgery, post-OR bed type (ICU/PACU/general ward), ASA score, number of home medications and renal function are among the most important features. The ASA score characterizes patient operative risk on a scale of 1–5, where 1 is a normal health condition and 5 is moribund^26^. It has already been shown that ASA is a predictor of medical complications and post-surgery mortality^28^. Our study showed that ASA is an important predictor of surgery duration as well. The age, BMI, and surgeon type (attendings vs. residents) features correlate to the important features aforementioned, which explains their exclusion by the uni-/multivariate analyses. For example, the patient’s age and BMI could implicitly influence the anesthesiologist’s estimate of ASA score and post-OR bed type. Note that most complex surgery procedures were performed by attendings. The surgical residents’ years of experience might affect OR times, but we did not have data to account therefore. The gender was pointed out as an unimportant feature for cardio-thoracic surgery duration by the Catharina anesthesiologists and indeed was statistically unsignificant in the univariate analysis.

The third key finding is that the ensemble model outperformed the LM, RF, and GB ML models with respect to reducing the surgeries “behind schedule” and increasing the surgeries “on time”. Since our data have complex underlying patterns, we stacked the linear (LM) with non-linear (RF and GB) model predictions into an ensemble model to get optimal performance. RF and GB represent the bagging and boosting ML techniques, respectively. Bagging is a variance reduction technique whereas boosting is a bias reduction technique and ensembling them improved accuracy while keeping data variance and bias low^29^.

The novel aspects of the models described in this paper are (1) inclusion of patient-/surgery-complexity characteristics in addition to the surgeon and surgery procedure; (2) use of features available in the scheduling phase of the surgery, prior to patient’s hospitalization, proving feasibility of good predictions without vital signs, lab results, and other monitoring data; (3) ensemble of linear and non-linear ML algorithms for best performance.

The OR is the major cost and revenue center for most hospitals and effective use of capacity resources can provide significant benefits as summarized by the following papers^30–33^. Parameters like surgery duration, post-OR bed type, and length-of-stay are essential for surgery planners and have been target variables of recent publications^34–36^. The models of elective and acute surgery duration we developed support surgery planners in a different way. The former is used in scheduling elective patients several days or weeks ahead of the patients’ hospitalization. The latter is used in elective patient rescheduling caused by an acute patient with a high medical urgency. The rescheduling can result in (1) exceeding the target surgery date of elective patients and (2) inefficient OR utilization. Hence, surgery planners need to decide whether any elective patient can be safely rescheduled and if yes which OR with elective surgery is the best to reschedule. These decisions are supported by the predictive models of acute surgery duration. Summing up, predictive models of elective and acute surgery duration facilitate complex patient scheduling with OR and ward occupancy rates close to the hospital’s KPIs. These models can also enhance decision-making processes elsewhere in the end-to-end chain of in-/out-patients services such as planning for intakes and patient preparation at the ward, or estimation of ICU bed capacity by predicting more accurately the patients’ OR in- and out-flows. It is worth mentioning that the surgery duration predictions need to be combined with OR cleaning time which is dependent on hospital-specific processes and can be derived from historical data.

Recently, there has been a substantial increase in AI research in medicine^37–40^, showing that healthcare professionals are most comfortable using AI for workflow tasks such as staffing and patient scheduling (64%), followed by clinical tasks such as flagging anomalies (59%), treatment plan recommendation (47%) and diagnosis (47%). The models described in this paper belong to the first group and may facilitate broader AI adoption by generating data-driven insights that show a positive impact on operational efficiency and capital investments.

Future work will investigate (1) the impact of the ML models on optimizing the surgery schedule and how it translates into more effective OR utilization and bed occupancy in the ICU and general ward, and (2) new predictive models for patients at high risk of complications that can generate meaningful alerts for long-lasting surgeries.

This study has some limitations. First, we analyzed only 2294 surgeries prior to the COVID-19 pandemic as shown in Fig. 2. The pandemic had affected not only surgery volume, but also surgery times and complexity. Patients’ complications due to postponed surgeries may lead to longer surgery duration. Back to “normal” OR schedule was seen mid-2022, which is why we could not use bigger and more recent data for analyses. Second, the need for predictive models tailoring on different levels - per hospital, per medical specialty, per surgeon, etc., limits the models’ scalability. While the model development methodology is reproducible, the model implementation is hospital-specific. Third, the new ML models are prone to drifting due to either a change in the relationship between the target and independent variables, missing input variables, or any other disruption. Changes in patient’s population, surgeon’s skills, surgeon’s fatigue, or surgery’s procedures over time are usually the root cause of the model’s drift leading to poor performance. Then, either retraining or including new features in the ML model accounting for disruptions are needed as a part of the ML models lifecycle. In contrast, the current model based on average surgery duration can quickly pick up upon data changes and might be used as a backup model. Despite the limitations, this study provides valuable insights into the shortcomings of the current models of surgery duration and how they can be overcome by leveraging advanced ML models.

In conclusion, ML technologies based on specific individual patient and surgery characteristics are a fundament of improved predictive models of cardio-thoracic surgery duration. These models are a stepstone towards data-driven capacity management. Surgery planners could benefit from these predictive models as a patient flow AI decision support tool to create an optimized surgery schedule for optimal OR utilization which is a prerequisite to effective capacity management.

## Methods

The methods used in the study are summarized according to the guidelines by Luo et al.^41^ for developing and reporting ML predictive models in biomedical research.

### Design

This was a retrospective predictive modeling study of surgery duration to estimate the OR time required for a patient’s cardio-thoracic surgery. The OR time was defined as a difference between the patient’s departure and arrival times in the OR, excluding the OR cleaning time. The study was approved by both—the Institutional Review Board (IRB) of the Catharina Hospital (nWMO-2020.165) and the Internal Committee for Biomedical Experiments (ICBE-S-000239) of Philips. An individual patient’s consent was waived due to the retrospective study design in accordance with the IRB rules of the Catharina Hospital.

### Study cohorts

In this study, we included cardio-thoracic surgeries performed in the Catharina hospital during the period Dec 2018—Feb 2020 on patients older than 18 years. The age restriction was related to the privacy requirement of the study protocol. We selected 15-month study period prior to COVID-19 to avoid the pandemic impact on surgery volume, surgery times, and medical urgency. Our focus on cardio-thoracic surgeries was driven not only by the main KPI of the Catharina hospital depicted in Fig. 1, but also by an additional Dutch healthcare regulations rule “the time on the waiting list for cardio-thoracic patients should be less than 7 weeks to prevent severe medical complications”^42^. This rule emphasizes the importance of tools for effective surgery scheduling, rescheduling, and OR utilization that this paper focuses on. We excluded surgeries according to the following criteria: (1) surgery duration longer than ave(cardio-thoracic surgery time)+ 3*sd(cardio-thoracic surgery time) ~8 h, which excluded 1% of all surgeries, (2) patients’ mortality during surgery, and (3) surgery performed by a surgeon with less than 10 surgeries in the study period. The rationale behind the third exclusion criteria was twofold. First, the surgery duration estimate by the current model was unreliable to compare with. Second, the number of surgeries per surgeon is too low for meaningful data analysis. Summing up, the three exclusion criteria were selected to clean up the surgery data set from outliers related to low surgery frequency per surgeon and long/short surgery duration, e.g., long duration due to complications and short duration due to mortality, that are unplannable. The resulting surgery data set comprised both elective and acute surgeries. The acute surgeries were performed within 24 h of patient admission according to the study definition. There were three study cohorts analyzed—overall, elective, and acute surgeries.

### Data sources and preprocessing

The primary data source for this study was the electronic health record (EHR) data repository of the Catharina Hospital. The data contain patient demographics, patient and surgery characteristics collected at the pre-operative screening, and real surgery duration recorded during inpatient encounters. Data over the period Dec 2018—Feb 2020 were extracted from the EHR using Microsoft SQL Server Management Studio (SSMS) 2018. Data management and deidentification were achieved through SSMS and pseudo-coding. All data were deidentified before analyses.

The data preprocessing included both data cleaning and data transformations. The data cleaning comprised removing duplicates, correcting out-of-range variables, and imputing missing values of the following categorical variables—BMI, ASA, Medications, and Creatinine (see Table 1). The imputation strategy was based on replacing missing values with “the most frequent category” or a newly created “unknown” category. The first data transformation consisted of converting discrete into categorical variables, where both medical and statistical rationale were involved. For example, we used well-established medical categories for BMI, ASA^26^, and Creatinine^43^, and underlying statistical distributions for age and number of home medications, see Table 1. The second data transformation consisted of performing clustering of surgical procedures. Due to the proprietary procedures codes used by the Catharina Hospital, we were not able to use the Clinical Classifications Software Refined (CCSR)^44^, which is based on standardized procedure coding systems like ICD-10-PCS. That is why hierarchical (data-driven) and medical (clinician’s expertise-driven) clustering was performed to group the procedure codes into categories meaningful for data analysis. The third data transformation was one-hot encoding of the categorical variables. It was necessary for linear ML models, which cannot take categorical input directly, in contrast to decision tree ML models.

### Predictive models development and evaluation

The current model deployed in the Catharina hospital uses the surgeon’s average procedure time of the last 10 patients as a prediction of surgery duration for the next patient having the same procedure. Further, the average surgery duration is sometimes manually corrected by the surgeons. The final estimate is referred to in this paper as a planned surgery duration by the current model used in clinical practice.

The new ML predictive models development went through the following steps: (1) data splitting, (2) feature selection, (3) model training, (4) model testing and evaluation, and (5) models ensembling.

In the first step, each of the three cohorts described above was randomly split into a training set (50%), a test set (20%), and a validation set (30%), which is common practice in data analysis. The splitting was “surgery”-aware but not “patient”-aware, i.e., the three sets were mutually exclusive with respect to surgeries but not patients with multiple surgeries. This splitting strategy might induce data leakage; however, the risk was minimal due to different procedures performed by different surgeons on patients with multiple surgeries (2% in the elective and 12% in the acute cohort). The training sets were used to develop predictive models including features selection and hyperparameters tuning based on a Random Grid Search K-fold Cross-Validation. We selected *K* = 10 for elective surgeries and *K* = 5 for acute surgeries since the latter data set was relatively small. Examples of RF hyperparameters tuned were the number of decision trees in the forest, maximum depth of the tree, the max number of features to consider at each split, and error measure to split on. The test sets were used to perform model stacking, i.e., fit ensemble models that combine the predictions of the different ML models developed on the training sets. In this way, the test sets became training sets for the ensemble models and that is why validation sets were needed to report model performance. So, the validation sets have been held out during the ML models development and were only used to evaluate the performance of the current model as well as all new predictive models as reported in Tables 4, 5 and Fig. 3.

In the second step, features were extracted from the EHR data related to patient cardio-thoracic surgeries. All these features were available at the point of surgery scheduling prior to patients’ hospitalization, and therefore lab results and monitoring data such as vital signs and ECG, were not available. The latter are expected to be strong predictors of real-time changes in surgery duration due to intra-operative complications which is a different user case than the patient’s surgery scheduling. We used a 2-step process for feature selection. First, we performed univariate inferential analysis to investigate the predictive power of each feature and dropped those that were not statistically significant predictors (*p* > 0.05). Second, we performed multivariate inferential analysis using Boruta algorithm^25^ based on RF ML technique that clusters features into important, tentative, and unimportant.

In the third step, we trained several predictive models using the features selected in the second step and multiple ML techniques—linear regression (LM), random forest (RF), and extreme gradient boosting (XGBoost; abbreviated as GB in this paper). RF and GB are non-linear models, very popular as algorithms of choice for many winning teams of machine learning competitions^45^.

In the fourth step, we used two well-known error metrics - RMSE and MAE - for the performance evaluation of regression models. Both metrics quantify the *delta* (*real-planned*) surgery duration, where *planned* refers to the surgery duration in hours predicted by the models, and *real* refers to the surgery duration in hours recorded in the Catharina EHR. In addition to RMSE and MAE, we defined customized error metrics in terms of surgeries “on time”, “behind schedule” and “ahead of schedule”. The categories “ahead of schedule” and “behind schedule” consist of all surgeries ahead and delayed, respectively, at least *t* min compared to their planned time. The category “on time” comprises all surgeries within the time range [*−t, t*] compared to their planned time. In our analysis we chose *t* = 10%*ave(cardio-thoracic surgery time) = 10%*(3 h 30 min) ~20 min. Furthermore, the category “ahead of schedule” had two subcategories with a time range of “more than 60 min” and “60–20 min” ahead of schedule, respectively. Similarly, the category “behind schedule” had three subcategories with a time range of “20–60 min”, “60–120 min”, and “more than 120 min” behind schedule, respectively. The time cut-offs of the subcategories corresponded to 30%*- and 60% * ave(cardio-thoracic surgery duration). We evaluated RMSE, MAE, and the customized errors on the current as well as new ML predictive models of surgery duration.

In the fifth step, we performed model stacking, i.e., the predictions on the test set made by the different models trained in the third step were used as features to fit a new model that we referred to as an ensemble model.

### Statistical analysis

The analysis of patients’ and surgeries’ characteristics by study cohort (elective vs. acute vs. overall) and by model development cohort (train, test, validation sets) were presented and summarized as means and standard deviations (SDs), or frequencies and percentages. Comparisons of normally/non-normally distributed continuous variables by cohorts were conducted using Student *t* tests/Mann–Whitney *U* tests, respectively. For categorical variables, Pearson Chi-square tests were used to examine the association between the cohorts. The significance level was set to *p* = 0.05. All data analyses were performed using the statistical software R, version 4.2.1^46^.

### Reporting summary

Further information on research design is available in the Nature Research Reporting Summary linked to this article.

### Supplementary information

Reporting Summary

## Data Availability

Restrictions apply to the availability of the data that support the findings of this study, since they were used under an IRB approval, and hence not publicly available.

## Code availability

The underlying code for this study performing the analyses on the training/test/validation data sets is not publicly available but may be made available to qualified researchers on reasonable request from the corresponding author.

## References

[1] Suzman R., Beard J. R., Boerma T., Chatterij S., Health in an ageing world–what do we know? *Lancet*, 10.1016/S0140-6736(14)61597-X (2015). 10.1016/S0140-6736(14)61597-X 25468156

[2] Blumenthal D, Davis K, Guterman S (2015). Medicare at 50—moving forward. N. Engl. J. Med..

[3] Daschle T., Frist W. For patients with multiple chronic conditions, improving care will be a bipartisan effort. *Health Affairs Blog*, 10.1377/hblog20170601.060354 (2017).

[4] Bodenheimer T. H., Chen E., Bennett H. Confronting the growing burden of chronic disease: can the U.S. health care workforce do the job? *Health Affairs*, 10.1377/hlthaff.28.1.64 (2009). 10.1377/hlthaff.28.1.64 19124856

[5] Scheffler R., Arnold D., Projecting shortages and surpluses of doctors and nurses in the OECD: what looms ahead. *Health Econ. Policy Law*, 10.1017/S174413311700055X (2019). 10.1017/S174413311700055X 29357954

[6] Peter G. Peterson Foundation. Per capita healthcare costs — international comparison based on financial data in, https://www.pgpf.org/ (2019).

[7] Rechel B., Funding for public health in Europe in decline? *Health Policy*, 10.1016/j.healthpol.2018.11.014 (2019). 10.1016/j.healthpol.2018.11.014 30509874

[8] Rumbold B., Smith J., Hurst J., Charlesworth A., Clarke A. Improving productive efficiency in hospitals: findings from a review of the international evidence. *Health Econ. Policy Law*10.1017/S174413311400022X (2015). 10.1017/S174413311400022X 25662195

[9] Atella et al. How health policy shapes healthcare sector productivity? Evidence from Italy and UK. *Health Policy*, 10.1016/j.healthpol.2018.10.016 (2019). 10.1016/j.healthpol.2018.10.016 30497784

[10] Carinci F. et al. Towards actionable international comparisons of health system performance: expert revision of the OECD framework and quality indicators. *Int. J. Qual. Health Care*, 10.1093/intqhc/mzv004 (2015). 10.1093/intqhc/mzv004 25758443

[11] van Houdenhoven M. Healthcare logistics: the art of balance, Erasmus University Rotterdam, The Netherlands, PhD thesis (2007).

[12] Drupsteen J. Treating planning flaws in patient flows, University of Groningen, The Netherlands, Ph.D. thesis (2013).

[13] Schneider A. J. Integral capacity management and planning in hospitals, University of Twente, Enschede, the Netherlands, Center for Healthcare Operations Improvement & Research, Ph.D. thesis (2020).

[14] Rutherford P. A., Provost L. P., Kotagal U. R., Luther K., Anderson A. Achieving hospital-wide patient flow. IHI White Paper. *Cambridge, Massachusetts: Institute for Healthcare Improvement.* 2nd edition, www.ihi.org; (2020).

[15] Vos L., Chalmers S. E., Dückers M. L., Groenewegen P. P., Wagner C., van Merode G. G., Towards an organisation-wide process-oriented organisation of care: a literature review. *Implement. Sci.* **6**, 10.1186/1748-5908-6-8 (2011). 10.1186/1748-5908-6-8 PMC3035025 21247491

[16] D’Andreamatteo A., Ianni L., Lega F., Sargiacomo M. Lean in healthcare: a comprehensive review. *Health Policy* **119**, 1197–209 (2015). 10.1016/j.healthpol.2015.02.002 25737260

[17] Gualandi R., Masella C., Tartaglini D. Improving hospital patient flow: a systematic review. *Buss. Process Manag. J.*10.1108/BPMJ-10-2017-0265 (2019).

[18] Ahlin Ph, Almström P., Wänström C. When patients get stuck: a systematic literature review on throughput barriers in hospital-wide patient processes. *Health Policy*, 10.1016/j.healthpol.2021.12.002 (2022). 10.1016/j.healthpol.2021.12.002 34969531

[19] World Health Organization. Global strategy on digital health 2020–2025, Available from: https://www.who.int/docs/default-source/documents/gs4dhdaa2a9f352b0445bafbc79ca799dce4d.pdf (2021).

[20] Fogel A, Kvedar J (2018). Artificial intelligence powers digital medicine. npj Digit. Med..

[21] Porter ME (2010). What is value in health care?. N. Engl. J. Med..

[22] Porter ME, Lee TH (2013). The strategy that will fix health care. Harv. Bus. Rev..

[23] Zanotto, B. S., Etges, APBDS, Marcolino, M. A. Z., Polanczyk, C. A. Value-based healthcare initiatives in practice: a systematic review. *J. Health Manag.*10.1097/JHM-D-20-00283 (2021). 10.1097/JHM-D-20-00283 PMC8423138 34192716

[24] Catharina Hospital Annual Report 2022, https://www.catharinaziekenhuis.nl/over-catharina/jaarverslagen/ (2022).

[25] Kursa M., Rudniski W. Feature selection with the Boruta Package. *J. Stat. Softw.* **36** https://www.jstatsoft.org/ (2010).

[26] ASA Physical Status Classification System. https://www.asahq.org/standards-and-guidelines/asa-physical-status-classification-system (2020).

[27] Rozario N., Rozario D. Can machine learning optimize the efficiency of the operating room in the era of COVID-19? *Can. J. Surg*. **63**, E527-E529 (2020). 10.1503/cjs.016520 PMC7747850 33180692

[28] Hackett N. J. et al. ASA class is a reliable independent predictor of medical complications and mortality following surgery. *Int. J. Surg*. 10.1016/j.ijsu.2015.04.079 (2015). 10.1016/j.ijsu.2015.04.079 25937154

[29] James G. et al. An introduction to statistical learning with applications in R, Springer, 2nd edn, https://www.statlearning.com/.

[30] Cardoen B., Demeulemeester E., Beliën J., Operating room planning and scheduling: a literature review. *Eur. J. Oper. Res.*10.1016/j.ejor.2009.04.011 (2010).

[31] Zhu, Sh et al. Operating room planning and surgical case scheduling: a review of literature. *J. Comb. Opt*. 10.1007/s10878-018-0322-6 (2019).

[32] Zenteno AC (2015). Pooled open blocks shorten wait times for nonelective surgical cases. Ann. Surg..

[33] Zenteno AC, Carnes T, Levi R, Daily BJ, Dunn PF (2016). Systematic OR block allocation at a large academic medical center: comprehensive review on a data-driven surgical scheduling strategy. Ann. Surg..

[34] Bartek M. A. et al. Improving operating room efficiency: machine learning approach to predict case-time duration. *J. Am. Coll. Surg*. **229**, 346–354.e3 (2019). 10.1016/j.jamcollsurg.2019.05.029 PMC7077507 31310851

[35] Fairley M., Scheinker D., Brandeau M. L. Improving the efficiency of the operating room environment with an optimization and machine learning model. *Health Care Manag. Sci*. **22**, 756–767 (2019). 10.1007/s10729-018-9457-3 30387040

[36] Meyfroidt G. et al. Computerized prediction of intensive care unit discharge after cardiac surgery: development and validation of a Gaussian processes model. *BMC Med. Inform. Decis. Mak*. **11**, 64 (2011). 10.1186/1472-6947-11-64 PMC3228706 22027016

[37] Raza M, Venkatesh K, Diao J, Kvedar J (2022). Defining digital surgery for the future. npj Digit. Med..

[38] The five transformations of future health, *e/MTIC White paper*, https://insights.hightechcampus.com/white-paper-future-health, March (2023).

[39] The Journey toward Insights at Scale for Health Care Providers, Briefing paper 2022, *Harvard Business Review*; https://hbr.org/sponsored/2022/10/the-journey-toward-insights-at-scale-for-health-care-providers.

[40] Future Health Index 2022. *Philips report*, https://www.philips.com/a-w/about/news/future-health-index.

[41] Luo W., Phung D., Tran T., Gupta S., Rana S., Karmakar C. Guidelines for developing and reporting machine learning predictive models in biomedical research: a multidisciplinary view. *J. Med. Internet. Res*. **18**, e323 (2016). 10.2196/jmir.5870 PMC5238707 27986644

[42] Dutch regulation of waiting time for healthcare services (in Dutch): Regeling Wachttijden en wachttijdbemiddeling medisch specialistische zorg - NR/REG-1823 - Nederlandse Zorgautoriteit. https://puc.overheid.nl/nza/doc/PUC_2034_22/2/.

[43] Nashef S (2012). EuroSCORE II. Eur. J. Cardiothorac. Surg..

[44] Healthcare cost and utilization project (HCUP), Clinical Classification Software Refined (CCSR), https://www.hcup-us.ahrq.gov/toolssoftware/ccsr/ccs_refined.jsp.

[45] Kaggle. Data Analytics Competitions; https://www.kaggle.com/competitions. (2021).

[46] R Core Team. (2015). R: A Language and Environment for Statistical Computing..

