# Supplementary material for: Predictive analytics for cardio-thoracic surgery duration as a stepstone towards data-driven capacity management
Source: NPJ Digit Med. 2023 Nov 7;6:205. doi: 10.1038/s41746-023-00938-0 (PMC10630382; doi:10.1038/s41746-023-00938-0)
Supplement: Supplementary file 1 — Reporting Summary [file 41746_2023_938_MOESM1_ESM.pdf]

nature research

Corresponding author(s): NPJDIGITALMED-06777
Mariana Nikolova-Simons

Last updated by author(s): Jul 24, 2023

Reporting Summary

Nature Research wishes to improve the reproducibility of the work that we publish. This form provides structure for consistency and transparency in reporting. For further information on Nature Research policies, see our Editorial Policies and the Editorial Policy Checklist.

Statistics

For all statistical analyses, confirm that the following items are present in the figure legend, table legend, main text, or Methods section.

n/a	Confirmed	
☐	☒	The exact sample size (n) for each experimental group/condition, given as a discrete number and unit of measurement
☐	☒	A statement on whether measurements were taken from distinct samples or whether the same sample was measured repeatedly
☐	☒	The statistical test(s) used AND whether they are one- or two-sided<br>*Only common tests should be described solely by name; describe more complex techniques in the Methods section.*
☐	☒	A description of all covariates tested
☐	☒	A description of any assumptions or corrections, such as tests of normality and adjustment for multiple comparisons
☐	☒	A full description of the statistical parameters including central tendency (e.g. means) or other basic estimates (e.g. regression coefficient) AND variation (e.g. standard deviation) or associated estimates of uncertainty (e.g. confidence intervals)
☐	☒	For null hypothesis testing, the test statistic (e.g. F, t, r) with confidence intervals, effect sizes, degrees of freedom and P value noted<br>*Give P values as exact values whenever suitable.*
☒	☐	For Bayesian analysis, information on the choice of priors and Markov chain Monte Carlo settings
☒	☐	For hierarchical and complex designs, identification of the appropriate level for tests and full reporting of outcomes
☒	☐	Estimates of effect sizes (e.g. Cohen's d, Pearson's r), indicating how they were calculated

Our web collection on statistics for biologists contains articles on many of the points above.

Software and code

Policy information about availability of computer code

Data collection	The code that supports the collection of this study data is based on SQL and EHR. No proprietary code or/and mathematical algorithms are used.
Data analysis	The code that supports the findings of this study is based on standard functions and packages available in R, which is an open source language and environment for statistical computing. No proprietary code or/and mathematical algorithms are used.

For manuscripts utilizing custom algorithms or software that are central to the research but not yet described in published literature, software must be made available to editors and reviewers. We strongly encourage code deposition in a community repository (e.g. GitHub). See the Nature Research guidelines for submitting code & software for further information.

Data

Policy information about availability of data

All manuscripts must include a data availability statement. This statement should provide the following information, where applicable:

- Accession codes, unique identifiers, or web links for publicly available datasets
- A list of figures that have associated raw data
- A description of any restrictions on data availability

Restrictions apply to the availability of the data that support the findings of this study, since they were used under an IRB approval, and hence not publicly available.

Field-specific reporting

Please select the one below that is the best fit for your research. If you are not sure, read the appropriate sections before making your selection.

☒ Life sciences ☐ Behavioural & social sciences ☐ Ecological, evolutionary & environmental sciences

For a reference copy of the document with all sections, see nature.com/documents/nr-reporting-summary-flat.pdf

Life sciences study design

All studies must disclose on these points even when the disclosure is negative.

Sample size	A retrospective predictive modeling study of surgery duration to estimate the OR time required for a patient's cardio-thoracic surgery. We included 2,294 cardio-thoracic surgeries performed in the Catharina hospital during the period Dec 2018 – Feb 2020 on patients older than 18 years.
Data exclusions	We excluded surgeries according to the following criteria: 1) surgery duration longer than 8 hours, which covers less than 1% of all surgeries, 2) patients' mortality during surgery, and 3) surgery performed by a surgeon with less than 10 surgeries in the study period.
Replication	The data analysis was performed using the open source statistical software R, and is completely reproducible.
Randomization	The complete data set was randomly split into a training set (50%), a test set (20%) and a validation set (30%), which is common practice in predictive modeling.
Blinding	n/a for retrospective predictive modeling study

Reporting for specific materials, systems and methods

We require information from authors about some types of materials, experimental systems and methods used in many studies. Here, indicate whether each material, system or method listed is relevant to your study. If you are not sure if a list item applies to your research, read the appropriate section before selecting a response.

Materials & experimental systems

n/a	Involved in the study	
☒	☐	Antibodies
☒	☐	Eukaryotic cell lines
☒	☐	Palaeontology and archaeology
☒	☐	Animals and other organisms
☒	☐	Human research participants
☐	☒	Clinical data
☒	☐	Dual use research of concern

Methods

n/a	Involved in the study	
☒	☐	ChIP-seq
☒	☐	Flow cytometry
☒	☐	MRI-based neuroimaging

Clinical data

Policy information about clinical studies

All manuscripts should comply with the ICMJE guidelines for publication of clinical research and a completed CONSORT checklist must be included with all submissions.

Clinical trial registration	nWMO-2020.165
Study protocol	Research Protocol General Human and Data Studies – Bench to Bedside – Capacity management; Predictive Analytics for Cardio-Thoracic Surgery
Data collection	See above
Outcomes	This manuscript focuses on evaluating the performance of the current surgery duration model used in clinical practices, and to develop and validate enhanced predictive models.
